# Drainage Continu par le Gaz Oxygene

**Published:** 1915-03

**Authors:** 

**Affiliations:** Assistant de Clinique Chirurgicale à Bruxelles


### DRAINAGE CONTINU PAR LE GAZ OXYGÈNE.

PAR

M. LE DOCTEUR RENOIRDRE,

*Assistant de Clinique Chirurgicale à Bruxelles.*

---

Le drainage dans la chirurgie abdominale à toujours été une des questions le plus discutées, chaque chirurgien ayant sa préference pour telle ou telle méthode.

J'estime à mon point de vue que les méthodes bien comprises et bien appliquées sont toutes bonnes.

Celle dont j'ai l'honneur de vous entretenir maintenant est entierement due à l'expérience de mon regretté Maitre le Dr. Jules Chiriar[1] de Bruxelles. Elle a surtout un cachet d'originalité, en ce sens qu'elle met en jeu plusieurs facteurs qui sont le drainage de la région abcédée, son antisepsie et sa réparation plus rapide. Je veux parler du drainage continu par le gaz oxygène. Le mot l'indique, c'est un drainage constant, permanent, qui une fois installé marche pour ainsi dire automatiquement, n'a besoin que de temps en temps d'une petite surveillance, celle de l'écoulement du gaz.

Celui-ci est fourni par une bonbonne (dans la pratique on se sert toujours d'une bonbonne contenant sous pression de quelques atmosphères un millier de litres d'oxygène) à laquelle est adapté un système de tubulure avec manomètre, terminé par un écrou de réglage permettant l'écoulement plus ou moins rapide, selon le cas. Une fois la poche abcédée ouverte et vidée, préalablement bien lavée, nous y plaçons deux drains de différent calibre. Le premier, adapté à la bonbonne, conduisant le gaz est de diamètre plus petit (environ No. 8) non troué sur les côtés et ne plonge qu'a environ un cm. du fond de la poche, le second, en contact direct avec le fond, est plus gros et troué comme un drain ordinaire, court sous les draps du patient et vient finir dans un récipiént rempli d'une solution antiseptique quelconque, amenant pus, sécrétions ou debris provenant de l'abcès.

Pour assurer un bon drainage plusieurs conditions sont requises. Tout d'abord vous devez, au sortir des drains de la paroi abdominale, un tant soit peu les comprimer par votre suture, ensuite les faire traverser plusieurs compresses coupées à leur milieu d'un orifice d'un diamètre plus petit que celui des deux drains de façon à les faire servir pour ainsi dire de tampons. Les drains mis en place et bien fixés, vous réglez votre écoulement du gaz. Ordinairement, je coupe le drain destiné au gaz, plus court, de façon à pouvoir le mettre en contact au moyen d'un tube en verre avec un autre tube

[1] Professeur de Clinique Chirurgicale.

en caoutchouc fixé à la bonbonne. Pour régler l'écoulement du gaz on enlève la première partie du drain et on la plonge dans un récipient rempli d'eau. En ouvrant la bonbonne, le gaz s'écoule en bouillonnant sous une trop forte pression, mais avec la vis de réglage vous diminuez la pression jusqu'à ce que la gaz s'écoule bulle par bulle.

C'est alors le moment de l'appliquer. Quelques moments après si votre drainage est bien fait et les drains bien saisis dans la plaie et par le pansement, vous voyez dans le vase récepteur le gaz s'écouler également bulle par bulle. Comme je l'ai dit plus haut il faut de temps en temps surveiller l'écoulement et s'assurer si le drain syphon n'est pas obstrué. S'il l'était une pression plus forte aurait vite raison de l'obstacle (coagulum sang, etc.). Cette recommandation est surtout faite pour le début du drainage. Ordinairement je ne le laisse en place plus longtemps que 48 heures. Le resultat est obtenu. Vous pouvez enlever les drains et refermer la plaie par quelques fils laissés à demeure. Ceci m'amène maintenant à vous parler de l'action tant mécanique que physiologique du gaz oxygène. L'écoulement du pus par le drainage n'est pas toujours chose facile c'est pourquoi, du reste, l'on place le drain dans les parties déclives de la région abcédée, de même que le patient est couché dans une position favorable. Avec le système de drainage continu nous y arrivons d'une façon sure et certaine. La pression exercée par le courant d'oxygène ne permet pas la stagnation du pus, ni son contact constant avec les tissus malades, c'est pour ainsi dire une poche qui doit se vider continuellement automatiquement. De ce fait vous obtenez rapidement l'assèchement des parties abcédées. En second lieu les observations faites par le Professeur Herman Jooris, Attaché à la Clinique Chirurgicale comme Directeur des Recherches bactèriologiques, ont montré l'action bienfaisante, rapide, éfficace de l'oxygène sur la phagocytose, sur la transformation des sérosités, sur son action tonique et sur son activité de réparations des tissus altérés. Du reste pour confondre certains confrères incrédules et montrer, preuves en mains, la grande éfficacité de l'action oxygénée sur la phagocytose, des expériences des plus concluantes sur des lapins et des cobayes injectés, ont été faites avec d'autres gaz ; tel que l'Hydrogène et l'acide carbonique. Les premiers effets, c'est à dire l'ecoulement mécanique du pus se faisaient sentir aussi bien qu'avec l'oxygène, mais l'aspect des liquides, des tissus, de même que l'examen bactériologique démontraient d'une façon irréfutable, l'inefficacité de ces deux gaz sur les éléments phagocytaires d'une part, et la supériorité non douteuse, tonique et vivifiante, du gaz oxygène d'autre part. Combien de fois aussi n'avons nous pas vu de ces volumineux abcès, profondément situés, drainés de cette façon, ne donner apres 24 heures que quelques légèrs suintements séreux qui nous permettaient d'enlever les drains, de refermer la plaie et d'avoir une réunion *per primam.* L'appel leucocytaire, à mon avis, joint à l'action mécanique de l'oxygène sont les grandes causes de réussite de cette méthode.

En effet, nous réalisons une antisepsie organique, nous mettons les tissus continuellement en contact avec une atmosphère régénératrice, en état de se défendre d'une façon magistrale et surtout ne subissant plus le voisinage de ce pus expulsé constament et facilement. Ici ouvrons une parenthèse et permettez-moi de vous citer quelques cas parmi tant d'autres. Je me souviens d'un cas d'appendicite phlegmoneuse avec gangrène de l'appendice, dont le résultat final à été merveilleux. Le malade était dans un état de septicèmie profonde, telle que quelquefois nous les voyions auparavant après les traitements médicaux infructueux, délire, pouls filant, rapide, facies tiré, température basse, ventre atonique, rétracté, enfin bref tous les symptômes d'un rapide dénouement. À l'ouverture, écoulement d'un pus fêtide, sanieux, rougeâtre fusant le long du colon ascendant. Après contre-ouverture lombaire j'applique le drainage continu. Contre mon attente le soir le malade revenait pour ainsi dire à la vie.

Le lendemain facies, pouls, température etaient meilleurs, tout indiquait déjà une desintoxication. La sérosité s'était transformée en pus de bonne nature.

Pendant 3 jours je lui ai laissé le drainage. Une quinzaine de jours après le malade quittait l'institut, la plaie en bonne voie de cicatrisation.

Laissez-moi vous citer un cas de phlegmon du ligament large. À l'examen on constatait une tumeur véritablement encastrée dans le bassin à gauche, non fluctuante, dure, ligneuse, fortement douloureuse. Incision mediane faite, impossibilité complête d'extirper la tumeur. J'incise le long de son grand axe et après nettoyage de la poche, drainage, suture des parois abcédées, et contre-ouverture latérale pour permettre la sortie des drains. Deux jours après je pouvais franchement les enlever.

La guerison à suivi le cours régulier d'une opération aseptique. Un autre cas encore d'abcès extrapéritonéal, suite d'accouchement ayant nécessité des manœuvres très laborieuses. Pratiquement le drainage se fait par la voie abdomino-vaginale et toujours demande un temps très long pour la guérison et l'asséchement parfait des parties abcédées. Après ouverture latérale et grand lavage, j'ai simplement drainé par l'abdomen sans ouverture vaginale, le résultat à été le même, aussi heureux aussi rapide que pour les autres interventions.

Par ces quelques cas qui me reviennnet à la mémoire, j'ai voulu vous montrer la grande simplicité d'application de cette méthode, non dangereuse, non douloureuse, et les beaux résultats que l'on peut obtenir.

